# Effect of removable functional appliances on mandibular length in patients with class II with retrognathism: systematic review and meta-analysis

**DOI:** 10.1186/s12903-017-0339-8

**Published:** 2017-02-01

**Authors:** Adriana Santamaría-Villegas, Rubén Manrique-Hernandez, Emery Alvarez-Varela, Claudia Restrepo-Serna

**Affiliations:** 1 CES-LPH Research Group, Universidad CES, Calle 10A. No. 22-04, Medellín, Colombia; 2 Universidad CES, Calle 10A. No. 22-04, Medellín, Colombia

**Keywords:** Malocclusion, Angle class II, Retrognathia, Meta-analysis, Orthodontics appliances, Functional

## Abstract

**Background:**

Orthopedic functional devices, are used to improve mandibular length in skeletal class II patients. However, the orthopedic functional device with the best effect to increasing the mandibular length, has not been identified before. Thus, the aim of the present investigation was to evaluate Randomized Controlled Trials (RCT), to determine the best functional appliance improving mandibular length in subjects with retrognathism.

**Methods:**

A systematic review and meta-analysis was performed, including studies published and indexed in databases between 1966 and 2016. RCTs evaluating functional appliances’ effects on mandibular length (Condilion-Gnation (Co-Gn) and Condilion-Pogonion (Co-Po)), were included. Reports’ structure was evaluated according to 2010 CONSORT guide. The outcome measure was distance between Co-Gn and/or Co-Po after treatment. Data were analyzed with Cochran Q Test and random effects model.

**Results:**

Five studies were included in the meta-analysis. The overall difference in mandibular length was 1.53 mm (Confidence Interval (CI) 95% 1.15–1.92) in comparison to non-treated group. The Sander Bite Jumping reported the greatest increase in mandibular length (3.40 mm; CI 95% 1.69–5.11), followed by Twin Block, Bionator, Harvold Activator and Frankel devices.

**Conclusions:**

All removable functional appliances, aiming to increase mandibular length, are useful. Sander Bite Jumping was observed to be the most effective device to improve the mandibular length.

## Background

The main reason for using functional removable appliances is to establish muscular balance, eliminate oral dysfunction, and allow a proper length of both the maxilla and the mandible [1]. Several studies have been performed in order to evaluate with different methods, the morphogenic mandibular changes, associated with the use of functional appliances to propulse forward the mandible. Frankel [2, 3], Bionator [4], Bass appliance [5], Herbst [6], Sander Bite Jumping [7], among others; could be found in the literature for this purpose. Individually, many studies have found changes in mandibular length and position, both in the sagittal and vertical plane [2–7]. However, when studies are grouped and analyzed together in systematic reviews and meta-analysis, controversies appear. Some reviews have found no statistically or clinically significant differences between groups treated with functional appliances and controls [8], while other authors have observed those differences to be statistically significant [9]. Additionally, studies have found other results for the treatment with functional appliances, such as secondary statistically significant mandibular elongation [10] and changes in the facial profile, due to incisal inclination [11].

Notwithstanding these results, differentiation of the effects of each type of functional device has not been assessed before. Only the SR published by Cozza et al. [8], evaluated the effect of different appliances on mandibular length. However, they included the Herbst appliance, even when its functional effects are different, due to its fixed mechanism of action. The combination of removable and fixed appliances in the systematic review, hampers a true interpretation of the results.

Due to the above explained premises, the purpose of this study was to evaluate the available scientific evidence in exclusively Randomized Controlled Trials (RCT), regarding the efficacy of each functional appliance found in the literature in improving mandibular length in subjects with class II malocclusion due to retrognathism.

## Methods

This SR was conducted in accordance with the guidelines of Transparent Reporting of Systematic Reviews and Meta-analyses (PRISMA-statement) (http://prisma-statement.org/) [12]. The protocol detailing the review method was developed “a priori” following initial discussion between members of the research team.

### Ethics and consent statement

Not applicable.

### Focused question

In patients with class II skeletal malocclusion, due to retrognathism; which of the different orthopedic functional devices, has the best effect aiming to improve the mandibular length?

### Search strategy and literature selection

On 12 January 2016, a systematic search of the medical literature was performed to identify all peer reviewed papers in the English and Spanish literature dealing with the efficacy of the functional appliances in improving mandibular length according to the search strategy described below. Inclusion in the review was based on the type of study, viz., RCTs describing the efficacy of functional appliances in improving mandibular length in children by the adoption of cephalometric measurements. Only those studies assessing the efficacy of removable functional appliances in participants between 6 and 18 years of age were included. In the case of investigations including samples of subjects aged under 6 or over 18, the studies were included in the review only if the children’s data could be extracted from the total sample. Studies performed on selected populations that combined fixed orthodontics or appliances with removable functional appliances, were excluded. All search steps and quality assessments were performed by a single author and then carefully checked by all the other authors to minimize bias during the review process. In case of disagreement, decision was reached by consensus of the majority of authors.

As a first step, a search in the National Library of Medicine’s PubMed Database, Cochrane, EMBASE and Lilacs databases was performed using the combination of the keyword terms (MeSH when applicable); malocclusion, angle class II, retrognathia, adolescent, child, orthodontic appliances and functional appliances were used to identify a list of potential papers to be included in the review. The search limits were set to papers in English or Spanish language, studies in humans, randomized clinical trial and dates of publication between years 1960 and 2016. From the list of citations, papers were screened on the basis of their title, and those ones clearly not pertinent to this review’s aim were excluded. The remaining citations were selected for abstract reading, and those which were of potential interest for this review, based on their abstract contents, were retrieved in full text. Then, the retrieved papers were read to decide on their inclusion/exclusion in the review. As further steps to expand the search, the Scopus and Google Scholar databases and four journal Publishers’ website search engines (Elsevier; Wiley-Blackwell; Quintessence Publishing; and Springer) were screened for additional papers of interest. Also, a search within the reference lists of the selected articles, a handmade search within relevant English and Spanish language peer reviewed journals in the pediatric dentistry and orthodontics fields, as well as within Universidad CES electronic library catalogues were performed.

This systematic review was registered in the international Prospective Register of Systematic Review (PROSPERO), registration number CRD42012002858. www.crd.york.ac.uk/prospero/ [Internet]. [cited 2012 sep 4].

### Quality assessment

Prior to evaluate the quality of each study, its structure of report was reviewed based on the 2010 CONSORT guidelines for RCT studies [13]. In this review, all items were assessed with respect to the effects of different functional appliances on the length of the mandible, according to the measurements Co-Gn and/or Co-Po. The methodological quality of the included studies was assessed according to the Cochrane systematic review guidelines and recommendations [14–16], and verified with the PRISMA checklist (Transparent Reporting of Systematic Review and Meta-Analyses) [12]. The cut-off criteria for selection of studies are contained in Table 1.

**Table 1:** Inclusion and exclusion criteria

Inclusion Criteria	Exclusion Criteria
Control group with untreated class II subjects. Treatment group: Patients with Class II malocclusion by retrognathism treated with any of the following appliances: Bionator, Twin Block, Activator, Sander Bite Jumping or Frankel.	Studies which evaluated mandibular length with point Ar.
Minimum 6 months in treatment.	Studies evaluating outcomes with MRI.
Articles in English or Spanish.	Studies which utilized the Herbst appliance. Insufficient data for analysis
Studies in humans	Treatment combined with extractions.
Studies in growing patients (aged between 6 and 18 years old).	Treatment combined with fixed appliances.
Studies evaluated with cephalometric radiographs that included mandibular length measurements using Co-Gn, Co-Po.	Surgical treatment
Randomized clinical trials (RCT) studies	

Appraisal of external validity was made according to the following checklist: (i) Sampling of subjects and assessment of sampling bias (i.e., failure to ensure that all members of the reference population have a known chance to be recruited in the study sample), (ii) description of mechanism for patient selection, (iii) Exclusion rate and withdrawals of patients for analysis. Appraisal of internal validity provides an assessment of: (i) adequate statistical analysis, (ii) description of control for confusing variables, (iii) blindness for the evaluation of results and (iv) the use of *p* values or confidence intervals to accompany the effect of the estimation measure. Each criterion was given a value of 1 when accomplished and 0 when failed. The quality of the studies was classified according to the sum of the scores in each criterion. Studies were rated as low (score less than 4), medium (scores between 4 and 5) and high (scores greater than 5).

### Data recorded from the selected studies

For each of the included studies, the following data/information were recorded: size and demographic features of the sample [age range (years), gender distribution (% of females)]; type of functional appliance used; duration of treatment (months), and cephalometric points used to measure mandibular length (Co-Gn or Co-Po).

### Statistical analysis

Statistical analysis was performed in order to estimate the pooled effect of the different functional appliances on the length of the mandible (Co-Gn or Co-Po), expressed as differences between treatment and control groups, regarding the mean mandibular length, calculated in millimeters.

The heterogeneity analysis between studies was performed with the Q Cochran Test [17, 18] using a critical value of *p* < 0.05, with the objective of determining the model of analysis by fixed or random effects.

Given that in all included studies, mandibular length was measured using the distance in millimeters between Co-Gn and Co–Po, the pooled effect combined was calculated as a weighted mean difference [18].

Due to the high quality of the study reported by Obrien et al. in 2003 [19], and that they reported the mean of length in the treatment and control group with the respective confidence interval; the standard deviation (SD) of the present meta-analysis, was calculated using the standard error of the mean sample of that study.

### Availability of data and materials

As this investigation is a Systematic Review and Meta-analysis, the data supporting the findings are available in the databases PubMed, Cochrane, EMBASE and Lilacs. The full-text of the articles were obtained in “Fundadores” library and the available electronic resources of Universidad CES.

## Results

### Literature search

The Medline, Cochrane, Embase and Lilacs databases search allowed identifying 50 publications, of which ten were excluded on the basis of title screening. Another 33 citations were excluded after abstract reading based on different reasons (i.e. measurements in the investigation didn’t include Co-Gn or Co-Po (*n* = 13); include only facial measurements (*n* = 5), control group was lacking (*n* = 5), measurements were based on superimposition of images (*n* = 1), measurements were based on changes on the implant placement (*n* = 2), studies evaluating psychosocial features (*n* = 2), control trials not randomized (*n* = 1), use of fixed appliances (*n* = 1), combination of extraoral devices and functional appliances (*n* = 2) and results based on cranial base results (*n* = 1)). Seven papers were selected for full-text retrieval.

**Fig. 1:**
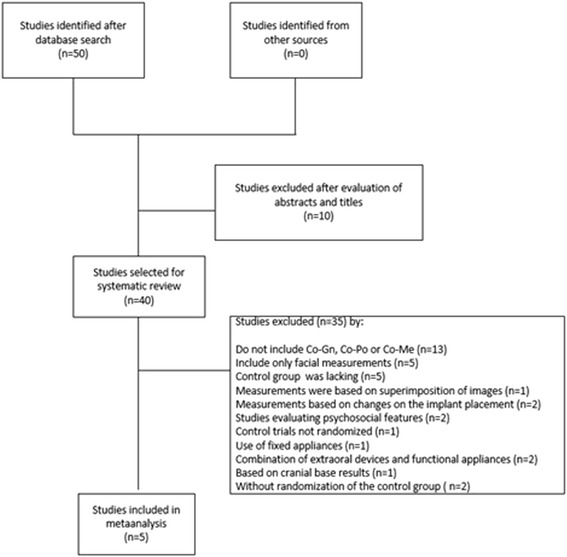
PRISMA Flowdiagram

Full-text reading allowed excluding two more papers, because they did not fullfill the inclusion criteria due to one main reason, viz., there was no random allocation of the control group. Sample was selected by convenience [20, 21]. Search expansion strategy retrieve no more citations. Hence, a total of five publications were found to be relevant to this meta-analysis assessment’s aim [7, 19, 22–24]. See Fig. 1 for a flowchart of the literature selection procedure.

### Methods of the included studies

An analysis of the methodological quality of the studies with the established criteria by the authors, indicated that two studies had a medium score [23, 24] and the other three [7, 19, 22] were classified as high quality.

The most frequent observed flaw, was that all studies lack of criteria for blindness for evaluation of results. Overall, studies presented acceptable samples sizes for identifying significant differences and sample selection criteria were reported in most of the studies. The four studies that reported the most adequate sample size calculation and inclusion and exclusion criteria, obtained the highest methodological score [7, 19, 22, 24]. One study reported withdrawals between 1% and 16% [22]. The article reported them adequately.

All studies presented suitable report of methods and statistical used to detect and analyze the effect of the intervention. Most of them used the error analysis method for radiographic measurements. Some studies reported the results with confidence intervals (CI) while others reported *p* values (Tables 2 and 3).

**Table 2:** Included studies

Appliance: sample/age (years)	Year of publication	Type of study	Measurement method	Type of Appliance	Duration of treatment	Cephalometric points for mandibular changes	Timing of treatment	Maturation analysis and method	Results conclusions of author
Tulloch [23]									
Bionator: 53 /9.4	1997	RCT	CXR^a^	Bionator	15 months	Mandibular length Co-Gn/Co-Pg+ (mm)	1 year before of peak	Carpal	Functional appliances produce mandibular changes with great variation
Control: 61/9.4					15 months				
Martina [7]									
Sander Bite Jumping: 23/10.9	2013	RCT	CXR^a^	Sander Bite Jumping	18 months	Mandibular length (Pg/OLp+ Co/OLp)	NA^b^	Cervical vertebrae	Treated individuals had a significant increase in mandibular length and molar relationship improvement
Control: 23/10.5					12 months	Mandibular length (Pg/OLp + Co/OLp)			
Illing [24]									
Twin block: 16/11.5	1998	RCT	CXR^a^	Twin Block	9 months	NA^b^	NA^b^	NA^b^	Both appliances demonstrated significant skeletal changes.
Bionator: 18/11.8				Bionator	9 months	Co-Gna(mm)			
Control: 20 /11.2					9 months	NA^b^			
Nelson [22]									
Harvold Activator: 12 /13	1993	RCT	CXR^a^	Classic activator	18 months	Co-Pog (mm)	NA^b^	NA^b^	No evidence supporting that functional appliances alter mandibular
Frankel: 13/13				Frankel	18 months	Co-Pog (mm)			
Control: 17 /13					18 months				
O Brien [19]									
Twin block: 73/9.7	2003	RCT	CXR^a^	Twin block	15 months	Mandibular length (Pg/OLp + Co/OLp)	Pre-peak	NA^b^	The Twin-block appliance resulted in substantial reduction in the overjets of children with Class II malocclusion.
Control: 74/9.8					15 months	(Pg/OLp + Co/OLp)			

+Measurement not clear in the text

^a^Cephalographic X-ray

^b^Information not available

**Table 3:** Quality of studies

RCT Studies	Explanation of calculation of sample size	Description of mechanism for patient selection	Explanation of the causes for withdrawal of patients	Statistical analysis adequate to the type of data	Description of the control for confusing factors	Blindness for the evaluation of results	Use of p values or confidence intervals	Evaluation of quality
Nelson 1993 [22]	yes	yes	Yes	yes	yes	no	Yes	High
Tulloch 1997 [23]	yes	yes	No	yes	yes	no	yes	Medium
Illing 1998 [24]	no	yes	Yes	yes	yes	no	yes	medium
O Brien 2003 [19]	yes	yes	Yes	yes	yes	no	yes	high
Martina 2013 [7]	yes	yes	Yes	yes	yes	no	yes	high

*RCT* Randomized clinical trial

### Treatment modalities

Regarding treatment modalities, Harvold Activator was used in one study [22], Twin Block in two studies [19, 22], type II Bionator was evaluated in two studies [23, 24], Frankel in one study [22] and Sander Bite Jumping in one study [7]. These data explain the fact that there are more comparisons than studies in the meta-analysis graphs.

**Fig. 2:**
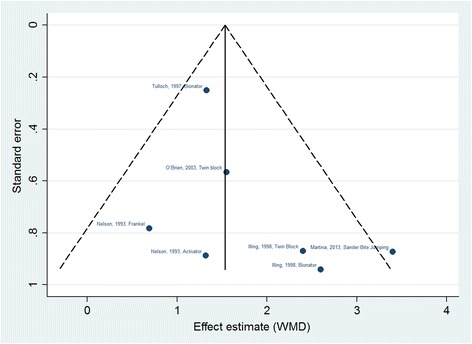
Funnel diagram of included studies. The funnel plot does not show the existence of publication bias among the included studies

Under the fixed effects model, the effect of functional appliances revealed a statistically significant increase in mandibular length measured as the distance between points Co-Gna or Co-Po (Fig. 2), with a difference in average mandibular length of 1.53 mm (CI 95% 1.15–1.92) with respect to non-treated control groups.

**Fig. 3:**
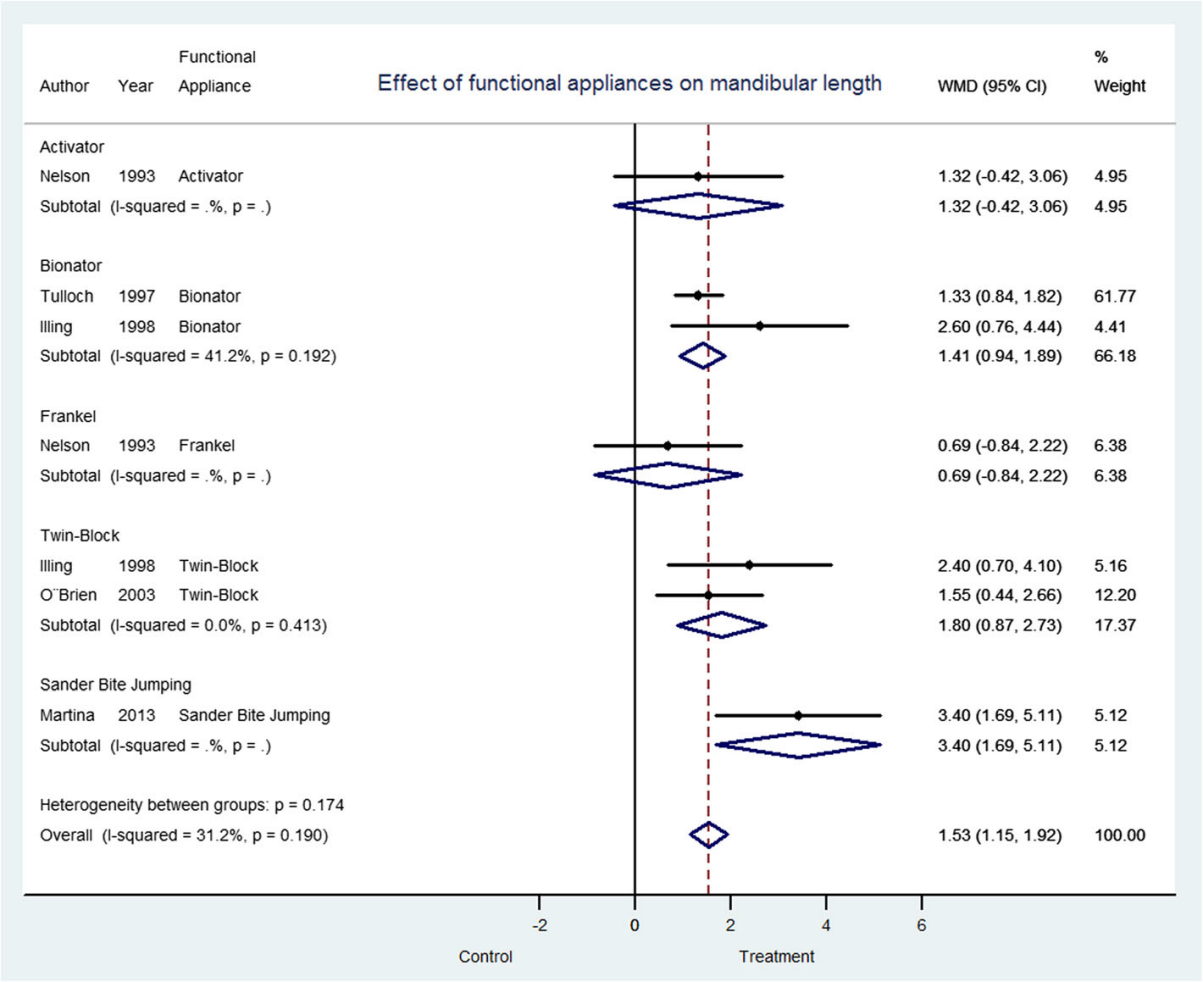
Effect of functional appliances on mandibular length depending on type of appliance. *WMD* weighted mean difference, *CI* confidence interval 95%

With respect to the efficacy of functional appliances, the Sander Bite Jumping reported the greatest increase in total mandibular length (3.40 mm; CI 95% 1.69–5.11), in comparison with other appliances which included, Twin Block (1.80 mm; CI 95% 0.87–2.73), Bionator (1.41 mm; CI 95% 0.94–1.89), Harvold Activator (1.32 mm; CI 95% −0.42–3.06) and Frankel (0.69 mm; CI 95% −0.84–2.22) (Fig. 3), although there were overlapping of the confidence intervals of the effects of Sander Bite Jumping, Twin Block and Bionator.

## Discussion

The use of removable functional orthopedic appliances in growing individuals with skeletal Class II, has demonstrated to be effective for the treatment of Class II malocclusion [2, 3, 6, 7, 25]. However, the evidence presents lack of consistency regarding the results shown both in studies [2, 3, 6, 7, 25] and systematic reviews [10, 11]. Additionally, a reliable differentiation of the effects produced by each type of functional appliance, has not been assessed before. Based on these premises, this systematic review evaluated the available scientific evidence in exclusively RCT, aiming to determine the efficacy of each type of functional appliance in improving mandibular length in subjects with class II malocclusion due to retrognathism. The five included investigations were analyzed in order to accomplish the objective.

All the studies included in this systematic review, are RCT. RCTs are the most rigorous way of determining whether a cause-effect relation exists between treatment and outcome and for assessing the cost effectiveness of a treatment, in this case different removable functional appliances. Other study designs, including non-randomized controlled trials, can detect only associations between an intervention and an outcome, but are not able to determine if the association was caused by a third factor linked to both intervention and/or outcome [26]. When these types of studies are included in systematic reviews, aiming to test the effect of a specific treatment (viz., the effects of orthopedic devices in increasing mandibular length), a true interpretation of the results is not possible.

Efforts were made in this investigation to increase the confidence of the results. Treatments which included teeth extractions, were excluded from the sample of this meta-analysis. The reason was that extraction of teeth, compensate the facial and skeletal profile [27]. Thus, is a confounding variable that could have interfered in our purpose of evaluating the effect of removable functional appliances in the increase-to-increase mandibular length.

The most common method to analyze mandibular length in the clinic, is the lateral cephalogram [11]. Actually, linear measurements of mandibular length have been observed to have high reproducibility between different times of measurement [28]. However, the main limitation to compare the measurements of mandibular length among the different studies, is that there is not consistency of measurement. Due to the above reasons, only the RCTs that used measurements in millimeters between Co-Gn and/or Co- Po were included, reducing the potential risk of measurement bias. As a strength of selecting specific points to evaluate the mandibular length, the combined estimate of the effect through weighting, was possible to be calculated. A previous systematic review used this method in order to assess the mandibular length after the use of orthopedic appliances, without determing the effect of each type of appliance [11].

The increase in mandibular length was greatest with the Sander Bite Jumping, followed by Twin Block, Bionator, and Harvold Activator, while the appliance that presented the least variation, was the Frankel. Assuming equal variances, the obtained error was so small to influence the combined estimation of the effect. These results contrast with those reported by Cozza et al. in 2006 [8], who found that the appliance giving the greatest variations was the Herbst followed by the Twin Block. The functional mechanism of action of the Herbst appliance is different from the mechanism of action of the removable functional appliances. Herbst is a fixed appliance, which promotes redirecting of maxillary growth, mesial movement of mandibular teeth and distal movement of maxillary teeth [29]. The different mechanism of action used by Herbst and the removable functional appliances, impedes a true comparison between them.

Clinical success cannot be measured only by mandibular length. Other factors should be considered, such as facial outcomes, solution of parafunctional oral habits and functional changes. This is a limitation not only of this review, but of almost all studies that evaluate treatment for mandibular propulsion. Two more limitations rised; first the studies by Nelson et al. [22], Tulloch et al. [23] and Martina et al. [7], describe the technique of taking the lateral cephalic x-ray in intercuspal position. The remaining studies (O’Brien et al. [19] and Illing et al. [24]), are not clear regarding the mouth position when taking the lateral cephalograms. However, in this systematic review and meta-analysis, we aimed to measure mandibular length, which is not affected by the position of the mandible in the cephalogram. Despite the technique to take the lateral cephalogram with mouth opening or intercuspal position, the measurement of mandibular length is stable.

Second, the age interval of the participants included in this investigation, is wide (6–18 years of age) and allows the result to be affected by the growth of the subjects. However, differentiating the effect of the evaluated removable functional appliances, on improving the mandibular length in children treated during the pre-pubertal stage and children treated in the pubertal spurt, increase the potential risk of reporting the influence of natural growth of the patients, as effect of the removable functional devices on improving mandibular length.

According to the results of the present investigation, orthopedic functional appliances are effective, independent of the type of appliance. The combined estimation of the effect evaluated by the fixed effect model, revealed a statistically significant increase in mandibular length of treated individuals. Mandibular growth was always greater in treated individuals regardless of the type of appliance used, indicating that functional appliances have a favorable effect on the correction of mandibular retrognathism. The Sander Bite Jumping was observed to be the most effective appliance aiming to improve the mandibular length, followed by the Twin Block. It is important to highlight that the results of the effect of the Sander Bite Jumping was based on only one article, whilst for the other devices, more evidence was presented. The clinical significance of the results has to be elucidated in future studies to weight the pertinence of the treatment with removable functional appliances.

## Conclusions

The findings of this meta-analysis, showed a slight increase in mandibular length (Co-Gn and/or Co-Po), after treatment either with Harvold Activator, Twin Block, type II Bionator, Frankel and Sanders Bite Jumping. The Sanders Bite Jumping reported the greatest results. The clinical relevance of this results have to be explored in further studies.

## Abbreviations

CI: Confidence interval
Co-Gn: Condilion-Gnation
Co-Po: Condilion-Pogonion
EBD: Evidence based dentistry
EBD: Evidence based dentistry
MA: Meta-analysis
PRISMA: Transparent Reporting of Systematic Reviews and Meta-analyses
PROSPERO: Prospective Register of Systematic Review
RCT: Randomized controlled trial
SR: Systematic review
WMD: Weighted mean difference

## References

[1] Woźniak K, Piątkowska D, Szyszka-Sommerfeld L, Buczkowska-Radlińska J (2015). Impact of functional appliances on muscle activity: a surface electromyography study in children. Med Sci Monit Int Med J Exp Clin Res.

[2] Cevidanes LHS, Franco AA, Gerig G (2005). Assessment of mandibular growth and response to orthopedic treatment with 3-dimensional magnetic resonance images. Am J Orthod Dentofacial Orthop.

[3] Cevidanes LHS, Franco AA, Gerig G (2005). Comparison of relative mandibular growth vectors with high-resolution 3-dimensional imaging. Am J Orthod Dentofacial Orthop.

[4] Araujo AM, Buschang PH, Melo ACM (2004). Adaptive condylar growth and mandibular remodelling changes with bionator therapy–an implant study. Eur J Orthod.

[5] Malmgren O, Omblus J, Hägg U, Pancherz H (1987). Treatment with an orthopedic appliance system in relation to treatment intensity and growth periods. A study of initial effects. Am J Orthod Dentofacial Orthop.

[6] Hägg U, Pancherz H (1988). Dentofacial orthopaedics in relation to chronological age, growth period and skeletal development. An analysis of 72 male patients with Class II division 1 malocclusion treated with the Herbst appliance. Eur J Orthod.

[7] Martina R, Cioffi I, Galeotti A (2013). Efficacy of the Sander bite-jumping appliance in growing patients with mandibular retrusion: a randomized controlled trial. Orthod Craniofac Res.

[8] Cozza P, Baccetti T, Franchi L, De Toffol L, McNamara JA (2006). Mandibular changes produced by functional appliances in Class II malocclusion: a systematic review. Am J Orthod Dentofacial Orthop.

[9] Marsico E, Gatto E, Burrascano M, Matarese G, Cordasco G (2011). Effectiveness of orthodontic treatment with functional appliances on mandibular growth in the short term. Am J Orthod Dentofacial Orthop.

[10] Zymperdikas VF, Koretsi V, Papageorgiou SN, Papadopoulos MA (2016). Treatment effects of fixed functional appliances in patients with Class II malocclusion: a systematic review and meta-analysis. Eur J Orthod.

[11] D’Antò V, Bucci R, Franchi L, Rongo R, Michelotti A, Martina R (2015). Class II functional orthopaedic treatment: a systematic review of systematic reviews. J Oral Rehabil.

[12] Moher D, Liberati A, Tetzlaff J, Altman DG (2009). Preferred reporting items for systematic reviews and meta-analyses: the PRISMA statement. BMJ.

[13] Schulz KF, Altman DG, Moher D (2010). CONSORT 2010 Statement: updated guidelines for reporting parallel group randomized trials. BMC Med.

[14] Wright RW, Brand RA, Dunn W, Spindler KP (2007). How to write a systematic review. Clin Orthop.

[15] Siwek J, Gourlay ML, Slawson DC, Shaughnessy AF (2002). How to write an evidence-based clinical review article. Am Fam Physician.

[16] Henderson LK, Craig JC, Willis NS, Tovey D, Webster AC (2010). How to write a Cochrane systematic review. Nephrol Carlton Vic.

[17] Higgins JPT, Thompson SG, Deeks JJ, Altman DG (2003). Measuring inconsistency in meta-analyses. BMJ.

[18] Petitti DB (2000). Meta-analysis, decision analysis, and cost-effectiveness analysis: methods for quantitative synthesis in medicine.

[19] O’Brien K, Wright J, Conboy F (2003). Effectiveness of early orthodontic treatment with the Twin-block appliance: a multicenter, randomized, controlled trial. Part 1: dental and skeletal effects. Am J Orthod Dentofacial Orthop.

[20] Dalci O, Altug AT, Memikoglu UT (2014). Treatment effects of a twin-force bite corrector versus an activator in comparison with an untreated Class II sample: a preliminary report. Aust Orthod J.

[21] Baysal A, Uysal T (2013). Soft tissue effects of Twin Block and Herbst appliances in patients with Class II division 1 mandibular retrognathy. Eur J Orthod.

[22] Nelson C, Harkness M, Herbison P (1993). Mandibular changes during functional appliance treatment. Am J Orthod Dentofacial Orthop.

[23] Tulloch JF, Phillips C, Koch G (1997). The effect of early intervention on skeletal pattern in Class II malocclusion: a randomized clinical trial. Am J Orthod Dentofac Orthop.

[24] Illing HM, Morris DO, Lee RT (1998). A prospective evaluation of Bass, Bionator and Twin Block appliances. Part I–The hard tissues. Eur J Orthod.

[25] Saikoski LZ, Cançado RH, Valarelli FP, de Freitas KM (2014). Dentoskeletal effects of Class II malocclusion treatment with the Twin Block appliance in a Brazilian sample: a prospective study. Dental Press J Orthod.

[26] Schulz KF, Chalmers I, Haynes RJ, Altman DG (1995). Empirical evidence of bias. Dimensions of methodological quality associated with estimates of treatment effects in controlled trials. JAMA.

[27] Booij JW, Goeke J, Bronkhorst EM, Katsaros C, Ruf S (2013). Class II treatment by extraction of maxillary first molars or Herbst appliance: dentoskeletal and soft tissue effects in comparison. J Orofac Orthop.

[28] Haas DW, Martinez DF, Eckert GJ, Diers NR (2001). Measurements of mandibular length: a comparison of articulare vs condylion. Angle Orthod.

[29] Pancherz H (1982). The mechanism of Class II correction in Herbst appliance treatment. A cephalometric investigation. Am J Orthod.

